# Assessing diet in a university student population: a longitudinal food card transaction data approach

**DOI:** 10.1017/S0007114520000823

**Published:** 2020-06-28

**Authors:** M. A. Morris, E. L. Wilkins, M. Galazoula, S. D. Clark, M. Birkin

**Affiliations:** 1 Leeds Institute for Data Analytics, School of Medicine, University of Leeds, Leeds LS2 9JT, UK; 2 Leeds Institute for Data Analytics, School of Geography, University of Leeds, Leeds LS2 9JT, UK

**Keywords:** Students, Diet, Dietary patterns, Big data, Transactions

## Abstract

Starting university is an important time with respect to dietary changes. This study reports a novel approach to assessing student diet by utilising student-level food transaction data to explore dietary patterns. First-year students living in catered accommodation at the University of Leeds (UK) received pre-credited food cards for use in university catering facilities. Food card transaction data were obtained for semester 1, 2016 and linked with student age and sex. k-Means cluster analysis was applied to the transaction data to identify clusters of food purchasing behaviours. Differences in demographic and behavioural characteristics across clusters were examined using *χ* ^2^ tests. The semester was divided into three time periods to explore longitudinal changes in purchasing patterns. Seven dietary clusters were identified: ‘Vegetarian’, ‘Omnivores’, ‘Dieters’, ‘Dish of the Day’, ‘Grab-and-Go’, ‘Carb Lovers’ and ‘Snackers’. There were statistically significant differences in sex (*P* < 0·001), with women dominating the Vegetarian and Dieters, age (*P* = 0·003), with over 20s representing a high proportion of the Omnivores and time of day of transactions (*P* < 0·001), with Dieters and Snackers purchasing least at breakfast. Many students (*n* 474, 60·4 %) changed dietary cluster across the semester. This study demonstrates that transactional data present a feasible method for dietary assessment, collecting detailed dietary information over time and at scale, while eliminating participant burden and possible bias from self-selection, observation and attrition. It revealed that student diets are complex and that simplistic measures of diet, focusing on narrow food groups in isolation, are unlikely to adequately capture dietary behaviours.

Starting university is an important time with respect to change in diet and wider lifestyle behaviours^(1)^. An unhealthy diet is a major risk factor for a variety of non-communicable diseases including type 2 diabetes, CVD and certain cancers^(2)^.

Food choice is a complicated behaviour associated with numerous factors, including culture, parental preferences, nutrition knowledge, stress levels and social class^(3–6)^. Women often display healthier habits compared with men, especially when diet is taken into account^(7)^. However, nutrition-related disorders or problems are also more common in women^(8)^. Diet quality has also been positively correlated with age^(9)^.

Studies indicate that first-year university students have a tendency towards an imbalanced diet irrespective of country of study^(7)^ or culture^(10)^. In a large study of 738 students at the University of Kansas^(11)^, for example, more than 69 % of students failed to meet the recommended serving of five portions of fruit and vegetables per d, and a similar proportion (67 %) did not meet the daily fibre recommendations (20 g/d).

There are numerous studies that have investigated student diets across several countries. Most are of cross-sectional design and use self-report measures of diet including 24 h recalls or FFQ to track the diet of students^(10,12–17)^. Some also use proxy measures of diet, such as fruit and vegetable consumption ^(11,18)^. Sample sizes vary widely from convenience samples of a couple of hundred^(19)^, through to tens of thousands in large cohort harmonisation or meta-analyses^(18,19)^. Where studies contain a longitudinal element, most capture only broad details about student diets, such as the number of meals and snacks per d^(19)^ or a brief FFQ containing twenty-two items, aggregated into six food groups^(16)^. These measures of diet prohibit detailed analysis of dietary consumption patterns. As a result of self-selection to participate in the studies and the participant burden associated with survey completion, risks of selection and attrition biases are high. As with most methods of dietary assessment, reporting bias is also likely^(20)^.

Transactional data from ready to eat food purchases could provide an objective measure of consumption and be easily monitored throughout the semester. Such data are not typically available. However, at the University of Leeds, students living in ‘catered’ halls of residence receive a ‘Refresh’ food card with credit for meals bought from the university refectory or coffee van. Data generated from these cards constitute a powerful tool to track student dietary behaviour.

The aims of this study are to (i) utilise food purchase transactions from all students living in catered halls of residence at the University of Leeds during their first semester to identify common dietary patterns, (ii) examine differences in demographic and behavioural characteristics across dietary patterns and (iii) investigate whether students maintain these patterns throughout the semester.

## Methods

### Study population

At the University of Leeds, first-year students living in on-campus catered halls of residences are provided with ‘Refresh’ food cards, which contain credit to cover two meals per d from Monday to Friday and brunch on weekends^(21)^. The cards can be used at the university refectory or coffee van and are included within students’ accommodation fees. Unused credit from 1 d is not carried over to the next.

During semester 1 of the 2016/2017 academic year, food cards were used by 835 first-year students. In October 2017 (1 year after the initial data generation), all of these students were provided with information about this study, proposing to anonymously use their first year, first semester, retrospective food card information, and given the opportunity to opt out of the study. Four students opted out. Students who were younger than 18 years (*n* 24) or older than 24 years (*n* 10) were also excluded from the study to prevent their potential identification due to low numbers. Two further students were excluded as they conducted fewer than one transaction per teaching week (one and two transactions over the whole study period, respectively), leaving a final sample of 795 students.

### Data sources

Food card data were extracted for semester 1 (12 September 2016–18 December 2016), covering the week before teaching began (Freshers’ week) to the week after teaching concluded. The food card data provided information on the location, date and time of each transaction, the name, quantities and costs of specific items purchased within each transaction and any promotional discounts applied (online Supplementary Table S1).

Daily food credit during the study period was £11·10 from Mondays to Fridays and £6·30 on Saturdays and Sundays. The university refectory was open 08.00–19.00 hours on weekdays and 10.00–14.00 hours on weekends. It served a range of hot and cold foods, with a daily-changing menu including breakfast (available 08.00—11.00 hours), hot and cold sandwiches, salads and a wide variety of cooked meals (example menu in online Supplementary Table S2). Snacks, cakes and hot and cold drinks were also available. The coffee van additionally served hot and cold drinks, pastries, cakes, filled baguettes and fresh bread and was open on weekdays 08.00–17.50 hours.

In order to explore demographic differences across dietary patterns, food card records were linked with university-held data on age and sex. Linkage was performed by an independent data services team and all data were anonymised prior to receipt by the research team. The anonymised data were screened prior to analyses, resulting in the exclusion of (i) 116 sales of an ‘empty cup’ and (ii) thirty transactions conducted at sites other than the refectory and coffee van (it was possible for students to ‘top up’ food cards to use in other food outlets on campus).

### Food classification

There were 651 unique items purchased using the food cards. These items were manually categorised according to the Department of Environment, Food and Rural Affairs (DEFRA) eating out food and drink codes^(22)^ (online Supplementary Table S3), in order to reduce the dimensions and optimise the clustering and its interpretation. The 651 items spanned twenty-one of the twenty-two DEFRA categories. There were no items in the DEFRA category ‘Alcoholic drinks’, as alcohol was not available for purchase using Refresh cards.

### Analysis and visualisation

All data analysis and visualisation were carried out using R Studio version 1.1.453 and R 3.5.0, using the ‘Riverplot’^(23)^, ‘Reshape2’^(24)^, ‘Plotrix’^(25)^, ‘Corrplot’^(26)^, ‘Chron’^(27)^ and ‘Ggplot2’^(28)^ packages.

#### Development of dietary patterns

Similar studies seeking to identify dietary patterns have used a variety of techniques such as principal component analysis, partial least squares regression and clustering algorithms^(29–31)^. k-Means clustering was used in our study, as this method is designed to group samples (in this case, students) into clusters that have similar features (in this case, purchasing behaviours). Furthermore, k-means has been shown to be more sensitive than other methods at detecting dietary patterns^(30)^.

Prior to clustering, the data were transformed to mitigate skewness and standardised to ensure equal weight for each variable. Specifically, for each student, the amounts spent on each food category were expressed as a proportion of that student’s total spend over semester 1 and then arcsine transformed. These transformed values were then standardised across each food type using *z*-scores. After transformation and standardisation, the k-means clustering algorithm was applied using a range of cluster numbers (1–20). The appropriate number of clusters was selected using a scree plot to identify the inflexion point and through consideration of the number of students per cluster, to ensure approximately equal cluster sizes.

#### Examining demographic and behavioural characteristics by cluster

We used *χ* ^2^ tests to explore differences in the distribution across dietary clusters of (i) student age (18, 19 or 20+ years), (ii) sex (male or female) and (iii) the time of day at which purchases were made.

### Diet change over time

In order to observe diet change over time, the sales for each student were further divided into three time periods. While the available data spanned 14 weeks, week 14 was a non-teaching week with a very low number of transactions (*n* 10) and was therefore excluded from this aspect of the analyses. Accordingly, the three time periods spanned weeks 1–5, 6–9 and 10–13, respectively.

For the purchases made by each student in each of these three time periods, their distances to each of the original cluster centres were calculated, using squared Euclidean distance, and each student was assigned to the cluster with the minimum distance. Cross-tabulations of the data were produced in order to follow the movement of students between clusters, with transitions also visualised using a Riverplot^(23)^.

## Results

### Study sample

The final sample included 795 students, who collectively conducted 107 723 transactions, spending £457 369 on 303 714 items over the semester (each transaction could include multiple items, e.g. sandwich and drink). Student-level demographic and transactional characteristics are reported in Table 1. The sample was predominantly aged 18 or 19, with more females than males.

**Table 1.** Demographic and transactional characteristics of our sample (Numbers and percentages; mean values and standard deviations)

	*n*	%
Sex		
Male	337	42·4
Female	427	53·7
Unknown	31	3·9
Age (years)		
18	392	49·3
19	221	27·8
20–24	153	19·2
Unknown	29	3·6
Transactional information	Mean	sd
Transactions per student over period (*N*)	135·5	40·9
Transactions per student per week (*N*)	10·9	4·5
Money spent per student over period (£)	575·26	113·92
Money spent per student per week (£)	46·43	14·66

*n*, Number of students; *N*, number of transactions.

Proportional spending per food group remained largely stable over the term (online Supplementary Fig. S1), with the exception of week 1 (Freshers’ week) and week 14 (the week after teaching concluded). There was also a notable increase in spending on ‘other food products’ in week 13 (the final week of teaching). Across the twenty-one DEFRA food groups, students spent the most money on ‘meat and meat products’ (£74 785), ‘soft drinks’ (£68 054) and ‘sandwiches’ (£46 301) and the least money on ‘yogurt s and fromage frais’ (£2282), ‘breakfast cereals’ (£3002) and ‘soups’ (£4083).

### Dietary clusters

Examination of the scree plot (online Supplementary Fig. S2) identified seven dietary clusters, summarised in Table 2 and illustrated using radial plots in online Supplementary Fig. S3–S9. The clusters were ranked for healthfulness based on food variety and the prominence of fruits, vegetables and salads within each pattern (online Supplementary Table S4). This provided a crude indication of the healthfulness of each cluster, used only to order clusters in tables and figures. It should not be taken as a holistic or accurate description of diet quality as there was insufficient information to calculate validated diet quality scores.

**Table 2.** Summary of dietary patterns, derived from data in the radial plots provided at online Supplementary Figs. S3–S9 (Numbers and percentages)

Cluster name	Rank*	Typical purchasing pattern	Cluster size	
			*n*	%
Vegetarian	1	High purchases: yogurt and fromage frais, breakfast cereals, salads	113	14·2
		Low purchases: meat and meat products, other food products, cheese and egg dishes or pizza		
Omnivores	2	High purchases: ice cream, desserts and cakes; breakfast cereals; fish and fish products	117	14·7
		Low purchases: confectionery, soft drinks including milk, sandwiches		
Dieters	3	High purchases: soups; rice, pasta or noodles; salads	122	15·3
		Low purchases: breakfast cereal; yogurt and fromage frais; ice cream, desserts and cakes		
Dish of the Day	4	High purchases: meat and meat products; Indian, Chinese or Thai food; other food products	126	15·8
		Low purchases: soups, biscuits, yogurt and fromage frais		
Grab-and-Go	5	High purchases: sandwiches; crisps, nuts and snacks; cheese and egg dishes or pizza	110	13·8
		Low purchases: soups; breakfast cereals; Indian, Chinese or Thai food		
Carb Lovers	6	High purchases: bread, cheese and egg dishes or pizza, ice cream, desserts and cakes	77	9·7
		Low purchases: salads, soups, yogurt and fromage frais		
Snackers	7	High purchases: confectionery; biscuits; crisps, nuts and snacks	130	16·4
		Low purchases: yogurt and fromage frais, salads, breakfast cereal		

*n*, Number of students.

* Rank: 1 = most healthy; 7 = least healthy (determined according to the prominence of fruits and vegetables and the variety of foods purchased).

### Demographic and behavioural characteristics of clusters

**Fig. 1.**
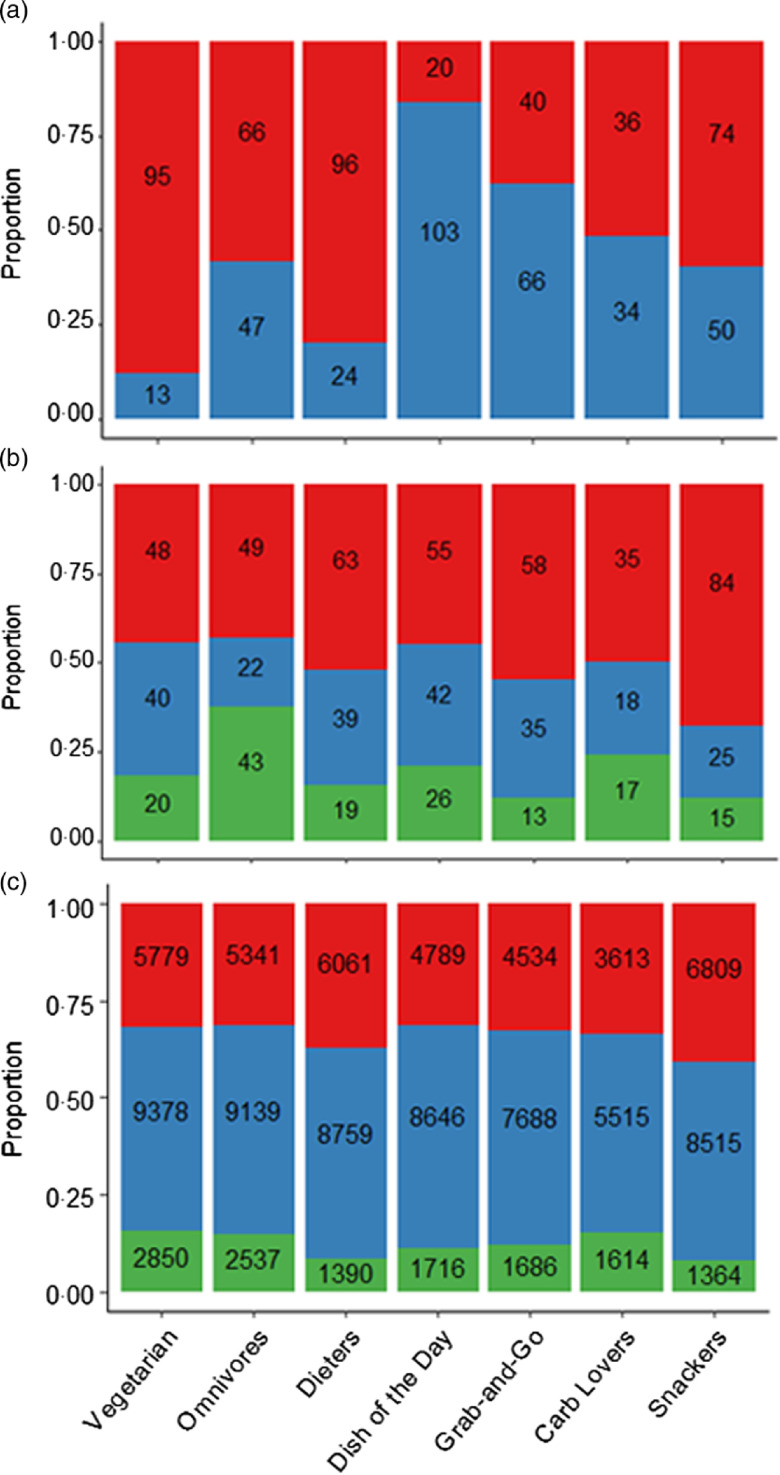
Distribution of sex (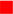, female; 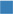, male), age (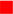, 18; 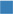, 19; 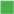, 20+ years) and time (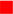, 17.00–19.00 hours; 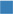, 11.00–17.00 hours; 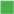, 08.00–11.00 hours) of transaction by cluster (panels (a)–(c), respectively). Labels on bars show numbers of students for panels (a) and (b), and numbers of transactions for panel (c). Panels (a) and (b) exclude students with unknown sex and age, respectively.

Fig. 1 shows demographic and behavioural characteristics of the clusters. Statistically significant differences in sex were revealed by *χ* ^2^ tests (*P* < 0·001), with women dominating the Vegetarian and Dieters clusters, age (*P* = 0·003), with over 20s representing a high proportion of the Omnivore cluster and time of transaction (*P* < 0·001), with Dieters and Snackers purchasing least between 08.00 and 11.00 hours (panels (a)–(c), respectively).

### Diet change through time

**Fig. 2.**
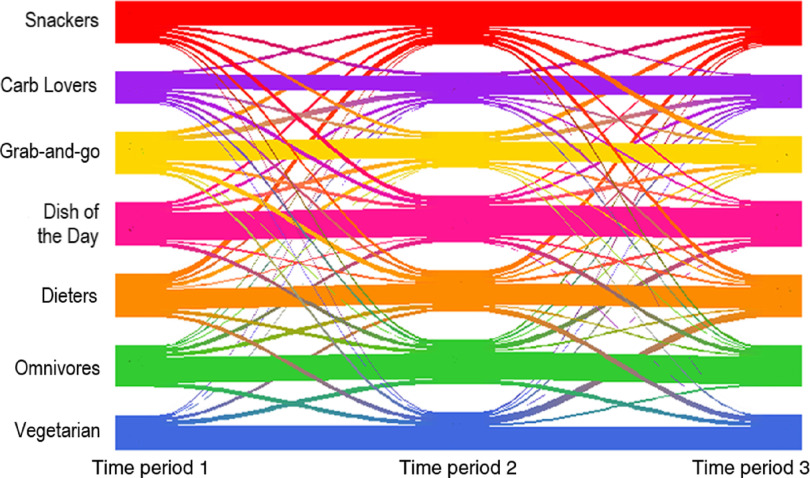
Riverplot showing the flow of students between dietary clusters at time periods 1–3.

There were 785 students with transactions in all time periods 1–3. Table 3 cross-tabulates students who remained in the same cluster or moved clusters between time periods. Fig. 2 displays these transitions using a Riverplot. A notable proportion of students (*n* 474, 60·4 %) changed dietary cluster across the semester (calculated using the sum of movements from time periods 1–2 and periods 2–3). The Grab-and-Go and Dieters groups were the most transitory. For example, 52·5 % of students in the Dieters cluster at period 1 transitioned to another cluster at period 2, and 50·4 % of the students in this cluster at period 2 were new students who had transitioned from another cluster in period 1. There were, however, no dominant patterns of movement between specific clusters. The highest number of students moving from one particular cluster to another was 35, which occurred from ‘Dieters’ to ‘Snackers’ (periods 1–2: nineteen transitions; periods 2–3: sixteen transitions). There is evidence that some students moved back to the same cluster which is highlighted when comparing time period 1 with time period 3 where only twenty-five students are observed to have transitioned from ‘Dieters’ to ‘Snackers’.

**Table 3.** Cross-tabulation of numbers of students within dietary clusters during time periods 1–3 (Numbers and percentages)

	Vegetarian	Omnivores	Dieters	Dish of the Day	Grab-and-Go	Carb Lovers	Snackers	% Moving out
Time period 1	Time period 2							
Vegetarian	69*	13	9	0	2	2	3	29·6
Omnivores	12	72*	12	11	3	4	3	38·5
Dieters	11	11	57*	4	11	7	19	52·5
Dish of the Day	0	11	5	79*	11	8	9	35·8
Grab-and-Go	9	2	15	10	56*	15	12	52·9
Carb Lovers	3	8	8	12	8	43*	8	52·2
Snackers	3	6	9	14	9	9	68*	42·4
% Moving in	35·5	41·5	50·4	39·2	44·0	51·1	44·3	
Time period 1	Time period 3							
Vegetarian	57*	9	18	1	4	4	5	41·8
Omnivores	13	66*	12	11	4	7	4	43·6
Dieters	14	6	52*	11	7	5	25	56·7
Dish of the Day	1	9	10	70*	13	11	9	43·1
Grab-and-Go	7	4	13	12	51*	17	15	57·1
Carb Lovers	4	11	7	7	8	41*	12	54·4
Snackers	4	9	8	15	15	10	57*	51·7
% Moving in	43·0	42·1	56·7	44·9	50·0	56·8	55·1	
Time period 2	Time period 3							
Vegetarian	67*	6	21	1	5	3	4	37·4
Omnivores	12	78*	5	14	3	6	5	36·6
Dieters	15	4	60*	8	6	6	16	47·8
Dish of the Day	1	12	7	85*	11	9	5	34·6
Grab-and-Go	4	3	5	7	54*	13	14	46·0
Carb Lovers	0	4	9	5	7	53*	10	39·8
Snackers	1	7	13	7	16	5	73*	40·2
% Moving in	33·0	31·6	50·0	33·1	47·1	44·2	42·5	

* Students who remained in the same cluster.

When change in pattern is stratified by sex, different patterns of change are observed, further highlighting the difference in behaviour between females and males. Please refer to online Supplementary Tables S5 and S6, Figs. S10 and S11 for these findings.

## Discussion

### Key findings

Our study employed a novel dataset to examine students’ food purchasing behaviours during an important life stage: the move to university. Using records of food purchases, obtained via student food cards, this study found seven distinct dietary patterns. Use of student food card data allowed detailed, objective measurement of food purchases over a sustained period, overcoming limitations and biases inherent in traditional research. Our findings provide a greater understanding of the dietary practices of students during a key transitionary period and help to identify potential groups of students to target in health-improvement interventions or in future research into underlying drivers for lifestyle behaviours.

### Overall dietary patterns

Many of the dietary patterns identified in this study comprised a mixture of ‘healthy’ and ‘unhealthy’ foods. For example, while the ‘Omnivorous’ group had particularly high purchases of desserts, they also consumed a wide variety of other foods, including high purchases of cereals, fish and vegetables which feature prominently in UK dietary guidelines^(32)^. This illustrates that student diets are not always either wholly ‘healthy’ or ‘unhealthy’ and that measurement of a small number of dietary components, as is common in the literature^(11,18)^, may be inadequate to capture the dietary practices of many students.

The above notwithstanding, it was possible to identify patterns of food purchasing that were comparatively less healthy. These included the Snackers, Carb Lovers and Grab-and-Go groups, which were all associated with limited food variety, low purchases of fruits, vegetables and salads, and high purchases of nutrient-poor and energy-dense foods. These groups collectively comprise nearly 40 % of students and present a potential target group for dietary interventions and further investigation.

One limitation of using data-driven techniques such as cluster analysis is that comparison with other literature is challenging. Nevertheless, two previous UK studies investigating the diets of university students^(33,34)^ and one investigating the diets of Irish adolescents^(30)^ have all observed dietary patterns similar to our ‘Snackers’ cluster, suggesting this may be a behaviour profile that transcends student/adolescent groups. Sprake *et al.* ^(33)^ also identified clusters similar to our Vegetarian and Dish of the Day clusters among 1448 UK university students, suggesting these may also be somewhat pervasive patterns. A scoping review of food choice amongst young adults in the USA identified similar general patterns, highlighting that snacking, rather than consuming three meals, is a popular behaviour in this age group, as observed in our Snackers and Grab-and-Go patterns. Additionally they observe that ‘healthy’ food items can be a driver of food choice in some, which we can see in the Vegetarian and Dieters clusters^(35)^.

### Behavioural and demographic variations

Our study cohort contained more females (53·7 %) than males. This aligns with university statistics indicating a higher percentage of female undergraduate admissions in 2016 (61·6 %)^(36)^. However, given that the difference between the proportion of males and females in our cohort is smaller than that of Leeds undergraduates more widely, results suggest that a higher proportion of males chose catered halls for their accommodation, although further investigation into the methods of assignment of accommodation would be required to confirm this.

Our findings broadly support past literature suggesting that dietary patterns differ with sex. Previous studies have found that females exhibit healthier dietary behaviours^(7)^ but are also more prone to nutrition-related disorders^(8)^. We found similarly complex relationships between sex and diet. For example, while females dominated the Vegetarian pattern (arguably the healthiest), there was also a high proportion of females in the Snackers pattern (arguably the least healthy), suggesting that females may tend towards dietary extremes. This is also supported by the dominance of females in the Dieters pattern, which was characterised by consumption of a very limited range of foods (predominantly soups).

Past research has found age to be positively correlated with diet quality^(9)^. Our study included students of a relatively narrow age range (18–24 years), yet still found differences in student ages across clusters. There was a dominance of older students in the Omnivores cluster and younger students in the Snackers cluster which partially supports the hypothesis that increasing age is associated with a healthier diet. However, the relationship was again complex. For example, there was a comparatively low proportion of older students in the ‘Vegetarian’ cluster which had the highest rank of healthfulness.

We investigated whether clusters differed in the time of day at which purchases were made. The Snackers and Dieters clusters tended to buy food items later in the day. Given that the Snackers were characterised by high spending on packaged foods, it is possible that these students are using up unspent credit for later consumption. This is in line with feedback from the catering marketing team, who felt purchases of snack food increased near to closing time. In contrast, it is somewhat surprising that the Dieters group also made a large amount of evening transactions, given that the foods purchased by this group tended to be ‘light’ meals typically associated with lunch (e.g. soups).

Comparatively few purchases were conducted between 08.00 and 11.00 hours. Skipping breakfast has been consistently associated with increased BMI and obesity risk among children and adolescents^(37)^. Our findings may therefore help explain the weight gain commonly observed among new university students^(38,39)^. However, we cannot rule out that students consumed breakfast at their accommodation or elsewhere, particularly as breakfast is often cheap and easy to prepare, requiring limited or no cooking skills and facilities, and therefore, students may save their food card credit for more costly/time-consuming meals.

### Change over time

Several studies have assessed dietary changes following the transition to university, with contradictory findings. For example, despite observing weight gain, Butler *et al.* ^(40)^ found that energy intake (assessed via FFQ) decreased among female freshmen students over the first 5 months of university, and Racette *et al.* ^(41)^ observed fried food intake decreased (again using questionnaires). These discrepancies are likely due to the inherent inaccuracies of traditional dietary assessment. Our study, which used objective data from food purchase cards, found that overall spending on DEFRA food categories was largely stable (excluding weeks 1 and 14, which were non-teaching weeks with fewer students present on campus). A notable exception to this rule was an increased spending on ‘other food products’ in the final week of term, attributable to purchases of Christmas dinners, which were only available in this week. Wansink *et al.* ^(42)^ found that unhealthy snack choices in a college cafeteria increased by 8 % in the last 2 weeks of term, and that this pattern reoccurred across subsequent terms. The authors hypothesised that assignment-related stress may be driving hedonic food purchases; however, we found no evidence of this in our data.

While spending on foods was stable when considering the sample as a whole, we found a high proportion of students moved between dietary clusters, suggesting dietary patterns do change at the individual level. Starting university represents a marked increase in dietary independence for many students^(1)^, and the fluidity of dietary patterns across the first semester may represent an exploratory phase, whereby students seek to establish new dietary habits. This period may therefore represent a prime opportunity for dietary intervention. Further research is needed over multiple semesters and years of university to establish longer-term dynamics of dietary behaviours.

Interestingly, the largest transition between clusters was from Dieters to Snackers. The Dieters cluster was also one of the most transitory clusters, suggesting this group of students may be following a limited variety, low-energy and ultimately unsustainable diet, and then reverting to other, often less healthy, dietary behaviours. This pattern of ‘yo-yo’ dieting has been associated with weight cycling and even weight gain^(43)^.

### Strengths and limitations

This study has several strengths. In contrast to traditional dietary studies, this study used objective transaction data at the individual level over a sustained period of time (14 weeks) to assess diet. Students did not know about the study at the time of data collection, eliminating observer bias. Additionally, while students had the opportunity to opt out, they did not actively need to sign up and commit their time to the research, limiting self-selection and attrition biases.

This study also has limitations. The food card data represent foods purchased, which we cannot be certain were consumed, although consumption was likely given these were ready to eat food purchases. The transactions did not contain information on all foods consumed in a day, and students likely consumed at least one additional meal elsewhere. The data also did not contain information on alcohol consumption, which is often a large part of student life in the UK^(19)^. These problems are exacerbated in that students did not typically spend their full credit every day, suggesting students may consume a considerable portion of meals outside of the university catering facilities. That said, this study does present an improvement over previous literature by objectively capturing a broader selection of foods purchased/consumed, over a longer period compared with traditional dietary research.

Food purchases were constrained by what was available, which was a broad but not limitless selection (online Supplementary Table S2). Having credits for catered food may also have influenced food choices compared with what would be eaten if meals were self-catered using students’ own budgets. For example, students may be more likely to consume cereal or toast for breakfast rather than a cooked breakfast due to speed and cost considerations. The findings of this study should therefore be generalised with caution.

We did not know the breakdown of students across the three halls of residences on campus and so were unable to account for differences across halls. That said, all halls were very close to the university refectory and coffee van (all within 150–300 m), and therefore, all students had similar access to the catered facilities.

Detailed information regarding the nutritional composition of purchased foods was unavailable. However, we did rank clusters based on the variety of foods purchased and the dominance of fruits and vegetables in the pattern, providing an approximate indicator of healthfulness which was useful for ordering clusters within tables and figures and spotting broad trends. Clustering was performed based on the amount of money spent, which is not necessarily indicative of amounts of foods consumed (in terms of kJ or g). However, clustering on price allowed us to account for promotions and to standardise students’ budgets for a fairer comparison.

In future research, it would be advantageous to link information on BMI for these students, using student medical practice records. However, this would be challenging from an ethical and governance perspective without informed consent and could reduce sample size and introduce bias.

### Conclusion

To our knowledge, this is the first time transactional student card data have been used to research health behaviours. This study demonstrates that data from food cards can be used as an alternative to traditional dietary assessment methods, which suffer from numerous limitations, as noted above. That said, a number of challenges were encountered in using these data. Firstly, ethical approval was challenging to obtain. While students agreed upon enrolment to the university that their data could be used in future research, they did not explicitly consent to participate in this study, and ethical approval was initially declined. Following appeal of the ethics decision, and assurance that no student would be identified, the ethics committee agreed an ‘opt-out’ as a compromise. Use of large consumer data in this way is novel, and some ethics committees may not yet be fully prepared to deal with it. A recent Delphi survey of experts in the field of obesity and big data called for ethical processes to be reviewed in this regard^(44)^. Linking the food card with university records on age and sex was challenging. Student identifiers within the university administrative systems were not compatible, and linkage had to be done via student emails, using an independent data services team in a secure ISO27001-accredited infrastructure, so that researchers were never exposed to student identifiers. Finally, as the food card data were managed by a third party, there was a fee of £750 + value added tax for the data extraction.

Insight generated by this research is now being used by the catering marketing team to help inform their health promotions to this group of students and others. There is potential for further health promotion beyond the university setting.

Despite the challenges, our novel data approach was shown to be achievable within typical budget and time constraints. Future research should investigate other sources of transactional data, such as supermarket loyalty cards, to allow access to different populations and increased scale.

## Availability of data and material

The data that support the findings of this study are available from a third-party company but restrictions apply to the availability of these data, which were used under licence for the present study and so are not publicly available.

## Supplementary material

For supplementary material accompanying this paper visit http://dx.doi.org/10.1017/S0007114520000823. click here to view supplementary material

## References

[1] Papadaki A , Hondros G , A Scott J , et al. (2007) Eating habits of university students living at, or away from home in Greece. Appetite 49, 169–176. 17368642 10.1016/j.appet.2007.01.008

[2] World Health Organization (2018) Non-communicable diseases factsheet. https://www.who.int/en/news-room/fact-sheets/detail/noncommunicable-diseases (accessed May 2019).

[3] Patrick H & Nicklas TA (2005) A review of family and social determinants of children’s eating patterns and diet quality. J Am Coll Nutr 24, 83–92. 15798074 10.1080/07315724.2005.10719448

[4] Wardle J , Parmenter K & Waller J (2000) Nutrition knowledge and food intake. Appetite 34, 269–275. 10888290 10.1006/appe.1999.0311

[5] Trudeau E , Kristal AR , Li S , et al. (1998) Demographic and psychosocial predictors of fruit and vegetable intakes differ: implications for dietary interventions. J Am Diet Assoc 98, 1412–1417. 9850109 10.1016/S0002-8223(98)00319-8

[6] Oliver G , Wardle J & Gibson EL (2000) Stress and food choice: a laboratory study. Psychosom Med 62, 853–865. 11139006 10.1097/00006842-200011000-00016

[7] Pearcey SM & Zhan GQ (2018) A comparative study of American and Chinese college students’ motives for food choice. Appetite 123, 325–333. 29337255 10.1016/j.appet.2018.01.011

[8] Arganini C , Saba A , Comitato R , et al. (2011) Gender differences in food choice and dietary intake in modern western societies In Public Health - Social and Behavioural Health, pp. 83–102 [ J Maddock , editor]. London: IntechOpen.

[9] Thiele S , Mensink GBM & Beitz R (2004) Determinants of diet quality. Public Health Nutr 7, 29–37. 14972069 10.1079/phn2003516

[10] Navarro-Prado S , Gonzalez-Jimenez E , Perona JS , et al. (2017) Need of improvement of diet and life habits among university student regardless of religion professed. Appetite 114, 6–14. 28315778 10.1016/j.appet.2017.03.017

[11] Huang TTK , Harris KJ , Lee RE , et al. (2003) Assessing overweight, obesity, diet, and physical activity in college students. J Am Coll Health 52, 83–86. 14765762 10.1080/07448480309595728

[12] Thongmuang P & Suwannahong K (2015) Health behaviours of undergraduate students in Suan Sunandha Rajabhat University. Procedia Soc Behav Sci 197, 973–976.

[13] Breitenbach Z , Raposa B , Szabó Z , et al. (2016) Examination of Hungarian college students’ eating habits, physical activity and body composition. Eur J Integr Med 8, 13–17.

[14] Stefan L , Cule M , Milinovic I , et al. (2017) The relationship between adherence to the Mediterranean diet and body composition in Croatian university students. Eur J Integr Med 13, 41–46.

[15] Alibabic V , Mujic I , Rudic D , et al. (2014) Assessment of diet quality and nutritional risks representation of University of Bihać. Procedia Soc Behav Sci 116, 2137–2140.

[16] Hilger J , Loerbroks A & Diehl K (2017) Eating behaviour of university students in Germany: dietary intake, barriers to healthy eating and changes in eating behaviour since the time of matriculation. Appetite 109, 100–107. 27864073 10.1016/j.appet.2016.11.016

[17] Seo Y & Je Y (2018) Disturbed eating tendencies, health-related behaviors, and depressive symptoms among university students in Korea. Clin Nutr Exp 19, 23–31.

[18] Steptoe A , Wardle J , Cui W , et al. (2002) Trends in smoking, diet, physical exercise, and attitudes toward health in European university students from 13 countries, 1990–2000. Prev Med 35, 97–104. 12200093 10.1006/pmed.2002.1048

[19] Serlachius A , Hamer M & Wardle J (2007) Stress and weight change in university students in the United Kingdom. Physiol Behav 92, 548–553. 17537466 10.1016/j.physbeh.2007.04.032

[20] Kipnis V , Midthune D , Freedman L , et al. (2002) Bias in dietary-report instruments and its implications for nutritional epidemiology. Public Health Nutr 5, 915–923. 12633516 10.1079/PHN2002383

[21] University of Leeds (2018) Refresh - great food at Leeds. https://gfal.leeds.ac.uk/refresh/ (accessed August 2018).

[22] Department for Environment, Food and Rural Affairs (2014) Household and eating out food & drink codes. https://assets.publishing.service.gov.uk/government/uploads/system/uploads/attachment_data/file/384778/familyfood-method-codes-11dec14.pdf (accessed March 2019).

[23] Weiner J (2017) Riverplot: Sankey or ribbon plots. R package version 0.6. https://CRAN.R-project.org/package=riverplot (accessed May 2019).

[24] Wickham H (2007) Reshaping data with the reshape package. J Stat Softw 21, 1–20.

[25] Lemon J (2006) Plotrix: a package in the red light district of R. R News 6, 8–12.

[26] Wei T & Simko V (2017) R package “Corrplot”: visualization of a correlation matrix (version 0.84). https://github.com/taiyun/corrplot (accessed May 2019).

[27] James D & Hornik K (2018) Chron: chronological objects which can handle dates and times. R package version 2.3-53. https://cran.r-project.org/web/packages/chron/index.html (accessed May 2019).

[28] Wickham H (2016) Ggplot2: elegant graphics for data analysis. New York: Springer-Verlag.

[29] Greenwood DC , Cade JE , Draper A , et al. (2000) Seven unique food consumption patterns identified among women in the UK Women’s Cohort Study. Eur J Clin Nutr 54, 314–320. 10745282 10.1038/sj.ejcn.1600941

[30] Hearty A & Gibney M (2011) Dietary patterns in Irish adolescents: a comparison of cluster and principal component analyses. Public Health Nutr 16, 848–857. 22014626 10.1017/S1368980011002473 PMC10271568

[31] DiBello JR , Kraft P , McGarvey ST , et al. (2008) Comparison of 3 methods for identifying dietary patterns associated with risk of disease. Am J Epidemiol 168, 1433–1443. 18945692 10.1093/aje/kwn274 PMC2727189

[32] Public Health England (2016) Eatwell guide. https://assets.publishing.service.gov.uk/government/uploads/system/uploads/attachment_data/file/528193/Eatwell_guide_colour.pdf (accessed March 2019).

[33] Sprake E , Russell J , Cecil J , et al. (2018) Dietary patterns of university students in the UK: a cross-sectional study. Nutr J 17, 90. 30290816 10.1186/s12937-018-0398-y PMC6172790

[34] Tanton J , Dodd LJ , Woodfield L , et al. (2015) Eating behaviours of British university students: a cluster analysis on a neglected issue. Adv Prev Med 2015, 639239. 26550495 10.1155/2015/639239 PMC4621329

[35] Powell PK , Durham J & Lawler S (2019) Food choices of young adults in the United States of America: a scoping review. Adv Nutr 10, 479–488. 31093651 10.1093/advances/nmy116 PMC6520045

[36] University of Leeds (2016) Student data 2016. https://equality.leeds.ac.uk/equality-data/student-data/student-data-2016/ (accessed January 2020).

[37] Szajewska H & Ruszczyński M (2010) Systematic review demonstrating that breakfast consumption influences body weight outcomes in children and adolescents in Europe. Crit Rev Food Sci Nutr 50, 113–119. 20112153 10.1080/10408390903467514

[38] Vadeboncoeur C , Foster C & Townsend N (2016) Freshman 15 in England: a longitudinal evaluation of first year university student’s weight change. BMC Obes 3, 45. 27826452 10.1186/s40608-016-0125-1 PMC5095959

[39] Vadeboncoeur C , Townsend N & Foster C (2015) A meta-analysis of weight gain in first year university students: is Freshman 15 a myth? BMC Obes 2, 22. 26217537 10.1186/s40608-015-0051-7 PMC4511069

[40] Butler SM , Black DR , Blue CL , et al. (2004) Change in diet, physical activity, and body weight in female college freshman. Am J Health Behav 28, 24–32. 14977156 10.5993/ajhb.28.1.3

[41] Racette SB , Deusinger SS , Strube MJ , et al. (2005) Weight changes, exercise, and dietary patterns during freshman and sophomore years of college. J Am Coll Health 53, 245–251. 15900988 10.3200/JACH.53.6.245-251

[42] Wansink B , Cao Y , Saini P , et al. (2013) College cafeteria snack food purchases become less healthy with each passing week of the semester. Public Health Nutr 16, 1291–1295. 23174136 10.1017/S136898001200328X PMC10271745

[43] Dulloo AG & Montani J-P (2015) Pathways from dieting to weight regain, to obesity and to the metabolic syndrome: an overview. Obes Rev 16, 1–6. 10.1111/obr.12250 25614198

[44] Vogel C , Zwolinsky S , Griffiths C , et al. (2019) A Delphi study to build consensus on the definition and use of big data in obesity research. Int J Obes 43, 2573–2586. 10.1038/s41366-018-0313-9 PMC6892733 30655580

