# Supplementary material for: Assessing diet in a university student population: a longitudinal food card transaction data approach
Source: Br J Nutr. 2020 Mar 5;123(12):1406–14. doi: 10.1017/S0007114520000823 (PMC7512144; doi:10.1017/S0007114520000823)
Supplement: Supplementary file 1 [file S0007114520000823sup001.docx]

## Supplement 1

Table 1. BEE-COAST framework summary of the food card data (Morris et al., 2018)

| **B**ackground | Key Features |
| --- | --- |
|  | For all first year students living in catered halls of residence at the University of Leeds, ‘Refresh’ food cards were credited each day with money to be used on student meals. Monday to Friday, students had a budget of £11.10 and at weekend £6.30 (as of 2016/17). Unspent credit was not carried over to the next day. These cards could be used at the University refectory or coffee van. |
|  | History & Purpose |
|  | First year students in catered accommodation are credited with money by the University to enable the University to provide catered halls of residence via its existing food retail facilities. This in turn provides students with greater choice and flexibility over their food purchases. It is also possible for students to add additional ‘top-up’ credit to their cards and to continue using them beyond their first year of studies, should they choose to add credit.  The ‘Refresh’ food card in general is available to any student or staff member at the University of Leeds to purchase food credits for use in University food outlets. From time to time, there are promotions only available to food card holders.  The Refresh card cannot be used for alcohol purchases. |
| **E**lements | **C**ontent |
|  | Student identifier, transaction identifier, total amount spent per transaction, items bought, cost per item, transaction time, transaction location, any promotional discounts applied to the transaction. |
|  | **O**wnership |
|  | The food card data are maintained by a third party company (MCR Systems Ltd.), employed by the University of Leeds. Acquisition of data requires payment to the third party for analyst time to generate the data extract. For the present study this cost was £750 + VAT. |
|  | **A**ggregation |
|  | Food card data are available at the level of food items purchased, and transactions (which may include multiple food items). These are linked to an individual student identifier. |
|  | **S**haring |
|  | Data are available on a case by case basis, subject to appropriate legal and ethical standards being met. |
|  | **T**emporality |
|  | The data extract used in this research is for 12 September 2016 to 18 December 2016. |
| **E**xemplars | Indicative use cases |
|  | This is the first use of food card data on individual-level food transactions to assess diet. |
|  | Foresight nodes |
|  | 6.4 Demand for health; 6.13 Market price of food offerings; 7.1 Force of dietary habits; 7.3 Tendency to graze; 7.4 Food exposure; 7.5 Food abundance; 7.7 Convenience of food offering; 7.8 Food variety; 7.11 Energy density of food offerings; 7.12 Fibre content of food offerings; 7.14 Demand for convenience; 7.16 Nutritional quality of food and drink. |

*VAT: value added tax.*

## Supplement 2

Table 2. Example refectory main meal menu adapted from University of Leeds (2019)

| **Category** | **MONDAY** | **TUESDAY** | **WEDNESDAY** | **THURSDAY** | **FRIDAY** |
| --- | --- | --- | --- | --- | --- |
| **HOT SANDWICH** | - Burger with cheese, jalapenos, relish, onion marmalade and tomato. - Sides: onion rings, spiral fries. | - Southern fried chicken burger - Sides: Monterey Jack cheese, beef tomato, garlic mayo, streaky bacon and chilli chips. | - Seared halloumi cheese with beetroot falafel, houmous in a quinoa burger bun. - Sides: salad, chilli jam, double fried wedges. | - Chilli beef enchiladas with cheesy nachos and salsa. - Sides: BBQ beans, corn on the cob, chilli chips. | - Jerk chicken pieces in toasted pitta - Sides: salad with chilli chutney, curly fries, blackened pepper and onions. |
| **DAILY SPECIALS** | **PIE DAY**   - Chicken Balti. - Cottage pie. - Cauliflower cheese - Sides: garden peas, chips, creamy mash. | **FISH DAY**   - Thai Fishcake with mint yogurt - Hot and spicy creel prawns. - Sides: wilted spinach and kale, roasted swede, chips, new boiled potatoes. | **LASAGNE DAY**   - Classic beef lasagne. - Sides: doughballs, sweetcorn, sauted vegetables, garlic and herb roasted potatoes, chips. | **ROAST DAY**   - Roast breast of turkey and sausage stuffing. - Sides: honey roasted root vegetables, broccoli florets, roasted potatoes, new boiled potatoes. | **CHIP SHOP DAY**   - Crispy battered cod - Jumbo battered sausage. - Sides: chips, mushy peas, curry sauce, baked beans. |
| **GREEN AND GO** | - Root vegetable & quinoa burger, with tomato, cheese and avocado. - Three bean and chickpea chilli. - Sides: roasted vegetable rice, cauliflower cheese, garden peas, parsley potatoes. | - Cheese and herb waffle with spicy Mexican beans. - Sweet potato, courgette and coconut curry. - Sides: boiled rice, Wilted spinach and kale, roasted swede, parsley potatoes. | - Spiced vegetable moussaka. - Homemade falafel balls with chilli ketchup. - Sides: spiced cous cous, sauted vegetables, baton carrots, parsley potatoes. | - Mushroom and lentil bake. - Roast vegetable cottage pie with veg gravy. - Sides: rice, honey roasted root veg, broccoli florets, parsley potatoes. | - Vegetable and apricot tagine. - Tomato, aubergine and spinach lasagne. - Roasted garlic and tomato cous cous. - Sides: roasted butternut and sweet potato, minted peas, parsley potatoes. |
| **PASTA** | - Penne pasta with pea, basil & mozzarella cheese. - Sides: dough balls, salt and pepper chips. | - Mushroom ravioli in a garlic and mushroom cream sauce. - Sides: dough balls, salt and pepper chips. | - Cheese tortellini in a cheese and chive sauce. - Sides: dough balls, salt and pepper chips. | - Vegetable chilli pasta. - Sides: dough balls, salt and pepper chips. | - Roasted root vegetable and mushroom risotto. - Sides: dough balls, salt and pepper chips. |
| **PIZZA** | - Spicy vegetable. - Hawaiian. | - Margherita. - BBQ chicken. | - Cheese & pesto. - Meat feast. | - Plum tomato and mozzarella. - Chorizo and mozzarella. | - Roast vegetable. - Pepperoni. |
| **SOUP** | - Vegetable. - French onion. | - Cream of butterbean. - Portuguese tomato and chicken. | - Tomato and basil. - Chinese chicken and sweetcorn. | - Spicy 3 bean. - Bacon and butternut. | - Super green vegetable. - Cream of chicken and bacon. |
| **STREET FOOD** | - Sweet & sour pork balls. - Sides: boiled rice, shrimp crackers, roti bread. | - Bulgogi beef. - Sides: boiled rice, shrimp crackers, flat bread. | - Baked satay cod. - Sides: boiled rice, shrimp crackers, naan bread. | - Deep fried crispy chilli chicken, - Sides: boiled rice, shrimp crackers, roti bread. | - Masala chickpea and vegetable curry - Sides: boiled rice, shrimp crackers, pitta bread. |
| **NOODLES** | - Chicken Strips, veg gyoza. - Spring roll. - Szechuan and veg wanton broth. | - Char sui pork, veg dumplings. - Satay chicken skewers. - Korean spicy and wanton broth. | - Szechuan beef, veg gyozas. - Chilli battered wings. - Pho and veg wanton broth. | - King prawns, neg dumplings. - Veg spring rolls. - Spicy Szechuan and Korean broth. | - Hoisin duck, veg gyoza. - Salt and pepper ribs. - Pho and veg wanton broth. |

The Refectory also serves a selection of cold sandwiches, salads, jacket potatoes, fruit, sweet and savoury snacks, cakes, and hot and cold drinks (non-alcoholic). Breakfast (comprising a selection of cereals, fruit, yoghurt and cooked foods) is served 8am-11am. All food is served ready to eat from hot and cold buffet bars and refrigerators, and students can mix-and-match items across the menus (e.g. a main from the ‘Pizza’ section, a side from the ‘Noodles’ section and a drink from the refrigerator).

## Supplement 3

Table 3. List of 22 DEFRA ‘eating out’ food categories, plus ‘Unusual foods’ grouping.

| **Classification** | | **DEFRA Examples*** |
| --- | --- | --- |
| 1 | Indian, Chinese or Thai food | Meat or fish-based curry with or without sauce  Vegetable or fruit-based curry  Dhal and dhal dishes  Samosas  Other Indian dishes  Indian breads  Indian buffet or shared meal  Chinese or Thai meat or fish-based dishes  Chop suey and fu yung dishes  Chinese or Thai vegetable-based main course dishes  Spring rolls  Other Chinese or Thai dishes  Chinese or Thai buffet or shared meal |
| 2 | Meat & Meat Products | Steak - without sauce (e.g. braised, sirloin)  Roast meat with sauce or gravy  Pork chops with sauce or gravy  Lamb chops with sauce or gravy  Spare ribs  Bacon, gammon or ham  All offal including liver, kidney, tongue  Chicken or turkey with sauce or gravy  Chicken or turkey in breadcrumbs or batter  Duck with sauce or gravy  Game with sauce or gravy  Burgers  Kebabs - all types  Sausages and sausage rolls  Hot dogs and sausage sandwiches  Meat pies (pastry or potato topped) and pasties  Meat and vegetable stews, casseroles or hotpots  Chicken or turkey stews, casseroles or hotpots  Meat-based oven baked dishes (e.g. lasagne, cannelloni, moussaka)  Paté |
| 3 | Fish and fish products | White fish - grilled, steamed, baked or boiled  White fish - fried (including in batter or breadcrumbs)  Trout, tuna and salmon  Herring, mackerel, sardines  Shellfish  Kippers and other smoked fish (e.g. smoked salmon)  Fish, processed, in breadcrumbs (e.g. fish fingers, fish cakes, scampi)  Fish burgers (in bun)  Fish based pie or other dish (e.g. paella, kedgeree, tuna pasta bake) |
| 4 | Cheese and egg dishes or pizza | Cottage cheese including with pineapple  Soft, continental or processed cheese (e.g. brie)  Cheddar, blue or other hard cheese and unspecified 'cheese'  Quiche and cheese pies or pasties  Other cheese dishes (e.g. Welsh rarebit, cheese and biscuits)  Pizza  Eggs - boiled or poached  Eggs - scrambled, fried, omelettes or unspecified 'egg'  Other egg dishes (e.g. egg mayonnaise) |
| 5 | Fresh and processed potatoes | Chips and French fries - from fast food outlet or served with meal  Potatoes - boiled, mashed, roast  Sautéed potatoes, potato croquettes, hash browns etc.  Baked or jacket potatoes  Other potato dishes (e.g. wedges, potato salad) |
| 6 | Vegetables | Lettuce and cress  Green vegetables (e.g. spinach, cabbage, sprouts)  Peppers - raw or cooked  Courgettes, marrow, aubergine, pumpkin, plantain, cucumbers  Peas and sweetcorn  Baked beans and other beans or pulses  Tomato - fresh, cooked or processed  Carrots  Onions - raw, cooked  Other root vegetables or tubers (e.g. turnip, parsnip, radish, beetroot)  Mushrooms  Mixed vegetables or unspecified 'vegetable'  Other vegetables (e.g. artichoke, asparagus)  Vegetables in batter or breadcrumbs (e.g. onion rings)  Onion and other vegetable bhajis and pakora  Vegetarian burger, bean burger, vegetarian sausage, nut roast  Oven-baked vegetable dishes (e.g. vegetable lasagne, cannelloni, moussaka)  Stuffed vegetables (e.g. stuffed pepper) and vegetable-based starter  Vegetable-based stews and casseroles and vegetable-based pies |
| 7 | Salads | Mixed salad, with or without dressing  Green salad, with or without dressing  Vegetable or fruit and nut salad  Pasta, rice, mixed bean or cereal-based salads  Meat salad (e.g. beef, lamb, chicken salads)  Fish salad (e.g. tuna, salmon salads)  Cheese salad including ploughmans  Egg salad  Other salads (e.g. Greek, Florida, Russian)  Salad buffet or buffet meal where items not specified |
| 8 | Rice, pasta or noodles | Fried rice and risotto  All cooked rice (e.g. boiled, pilau, savoury)  Pasta - not filled and plain noodles  Pasta - filled (e.g. ravioli, tortellini)  Noodles with meat, vegetables etc. |
| 9 | Soups | Meat & fish soups  Vegetable based soups  Chinese soups, consommé (e.g. meat, fish or vegetable) |
| 10 | Breakfast cereals | Muesli and oat crunch cereals  Other high fibre breakfast cereals (e.g. Allbran, Weetabix)  Sweetened breakfast cereals (e.g. Frosties, Sugar Puffs)  Hot breakfast cereals (e.g. porridge, Ready Brek)  Other breakfast cereals (e.g. Cornflakes, Rice Krispies, Special K) |
| 11 | Fresh and processed fruit | All citrus fruit (e.g. orange, grapefruit)  Bananas  Apples  Pears  Stone fruit (e.g. apricot, plum, peach, cherry, avocado)  Grapes  Soft fruit or berries (e.g. strawberries, blackberries)  Melon  Pineapple  Fresh fruit salad  Other fresh fruit (e.g. kiwi, passion)  Free school fruit  Dried fruit (e.g. sultanas, raisins)  Tinned, stewed, baked or processed fruit |
| 12 | Yoghurt and fromage frais | No examples provided |
| 13 | Bread | White bread (toasted or untoasted)  Brown or wholemeal bread (toasted or untoasted)  Rolls, baguettes etc. (white, brown or wholemeal)  Garlic bread  Croissant  Continental breads (e.g. pitta, ciabatta, focaccia)  Muffins, crumpets  Fried bread, including croutons  Other bread, rolls, toast, unspecified 'bread' etc. |
| 14 | Sandwiches | Meat-based sandwich  Chicken or turkey-based sandwich  Bacon and egg-based sandwich  Fish-based sandwich  Cheese-based sandwich  Egg-based sandwich  Vegetarian-based sandwich  Sweet-filled sandwich  Unspecified sandwiches |
| 15 | Other food products | Cheese or cream-based sauce (e.g. carbonara, cauliflower cheese)  Meat-based sauce (e.g. bolognese, chilli con carne)  Fish or seafood-based sauce  Tomato-based sauce containing vegetables, including ratatouille  Other savoury sauce  Sweet sauce (e.g. syrup, treacle, chocolate sauce)  Fruit or vegetable-based condiments  Other condiments or sauces  Salad dressings and dips  Mayonnaise  Coleslaw  Fruit filling (e.g. peaches for pancakes)  Vegetable filling  Cheese filling including cheddar cheese, cottage cheese  Fish-based filling (e.g. tuna mayonnaise)  Butter and margarine  Jam, marmalade and honey  Cream - single, double, sour etc.  Custard  Sugar (as an addition to tea, coffee etc.)  Commercial baby food in a jar or can  Yorkshire puddings and dumplings  Unspecified meal (e.g. 'meal', 'school meal' or 'meal at work)' |
| 16 | Beverages | Coffee (e.g. black, white, latte)  Tea (e.g. white, black, herbal, fruit)  Hot chocolate or cocoa |
| 17 | Soft drinks including milk | Mineral water  Soft drink (including carbonates and still)  Pure fruit juices  Vegetable juices (e.g. tomato juice, carrot juice)  Milk as a drink  Milk on cereal  Milkshake and flavoured milk  Free school milk |
| 18 | Alcoholic drinks | Spirits  Liqueurs  Cocktails  Spirits or liqueurs with mixer e.g. gin & tonic, Bacardi & coke  Table wine  Sparkling wines (e.g. Champagne) and wine with mixer (e.g. Bucks Fizz)  Fortified wine (e.g. sherry, port, vermouth)  Cider or perry  Alcoholic soft drinks (alcopops), and ready-mixed bottled drinks  Bitter  Lager or other beers  Round of drinks, alcohol not otherwise specified |
| 19 | Confectionery | Solid, unfilled chocolate bars and sweets  Filled chocolate-coated bars and sweets (e.g. Mars, Snickers, Minstrels)  Single chocolate (after dinner)  Chewing gum and bubble gum  Mints (e.g. Polo, Extra Strong)  Boiled sweets, jellies  Toffee or fudge (e.g. chocolate éclairs, caramels)  Pick 'n' mix, nougat, liquorice and other sweets |
| 20 | Ice cream, desserts and cakes | Ice cream  Iced lollies and sorbets  Doughnut  Cream pastries (e.g. chocolate éclairs, profiteroles)  Cream sponge or gateau  Rich chocolate cake or chocolate gateau  Fruit and other pies or pastries  Fruit cake  Other sponge cakes or desserts  Custard desserts or sweet soufflé  Meringue desserts including pavlova  Cheesecake  Fool, trifle and mousse desserts  Jelly  Milk and rice puddings including tapioca, semolina  Other cakes and desserts, unspecified  Waffles and pancakes  Teacakes, scones, currant buns, iced buns |
| 21 | Biscuits | Fully-coated chocolate biscuits or wafers  Sweet biscuits including half-coated chocolate biscuits  Cereal bars and cereal based cakes  Savoury biscuits |
| 22 | Crisps, nuts and snacks | Nuts, nut products and seeds  Potato crisps or snacks including unspecified 'crisps', prawn crackers  Cornsnacks, based on maize  Wheat-based savoury snack  Popcorn  Other savoury snacks (including hors d'oeuvres) |

** All examples are those provided in the DEFRA ‘Household and Eating Out Food & Drink Codes’ (DEFRA, 2014).*

## Supplement 4


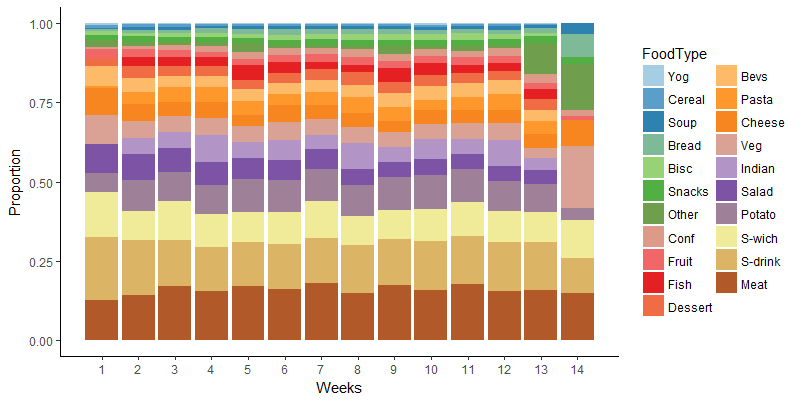


Figure 1. Money spent per food category per week.

*Weeks 1 and 14 were non-teaching weeks. Week 1 = 668 transactions, Weeks 2-13 = mean 8923 transactions (range: 7303-9519), Week 14 = 10 transactions.*

*DEFRA codes have been abbreviated as shown: Indian: Indian, Chinese or Thai food; Meat: Meat & Meat Products; Fish: Fish and fish products; Cheese: Cheese and egg dishes or pizza; Potatoes: Fresh and processed potatoes; Veg: Vegetables; Pasta: Rice, pasta or noodles; Cereal: Breakfast cereals; Fruit: Fresh and processed fruit; Yog: Yoghurt and fromage frais; S-drink: Soft drinks including milk; Conf: Confectionery; Dessert: Ice cream, desserts and cakes; Bisc: Biscuits; Snacks: Crisps, nuts and snacks.*

## Supplement 5


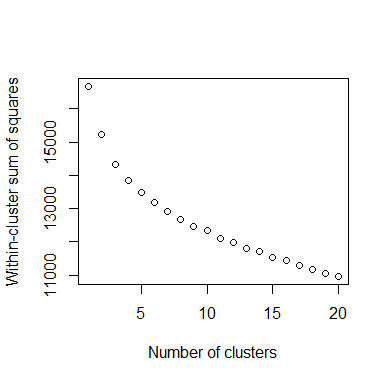


Figure 2. Scree plot showing within-cluster sum of squares for cluster numbers 1-20.

## Supplement 6

Radial plots illustrate the content of each of the dietary patterns. The 22 food groups are presented around the outside of the plot. The band with value 1, represents average of purchases in this cohort of students. Foods with value less than 1 are purchased less than average in this cluster and foods over 1, more than average.

### Vegetarian


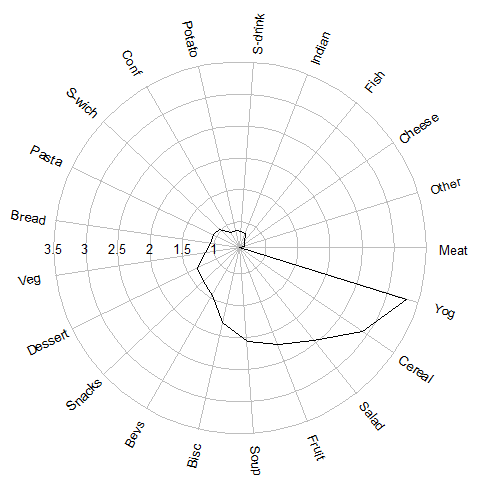


Figure 3. Radial plot showing amount spent per food group for the ‘Vegetarian’ pattern relative to the mean spending per food group across all clusters (index 1 represents the mean spend for a given food group).

*DEFRA codes have been abbreviated as shown: Indian: Indian, Chinese or Thai food; Meat: Meat & Meat Products; Fish: Fish and fish products; Cheese: Cheese and egg dishes or pizza; Potatoes: Fresh and processed potatoes; Veg: Vegetables; Pasta: Rice, pasta or noodles; Cereal: Breakfast cereals; Fruit: Fresh and processed fruit; Yog: Yoghurt and fromage frais; S-drink: Soft drinks including milk; Conf: Confectionery; Dessert: Ice cream, desserts and cakes; Bisc: Biscuits; Snacks: Crisps, nuts and snacks*

### Omnivores


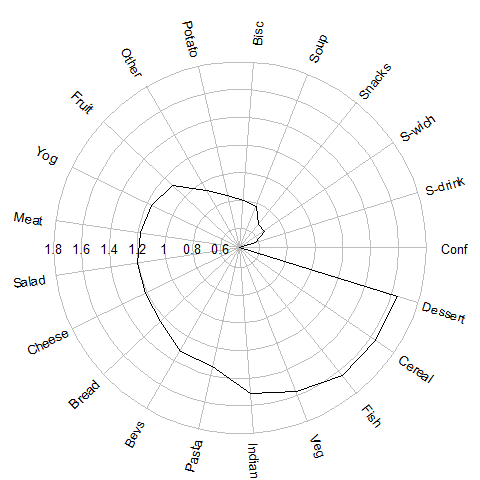


Figure 4. Radial plot showing amount spent per food group for the ‘Omnivores’ pattern relative to the mean spending per food group across all clusters (index 1 represents the mean spend for a given food group).

*DEFRA codes have been abbreviated as shown: Indian: Indian, Chinese or Thai food; Meat: Meat & Meat Products; Fish: Fish and fish products; Cheese: Cheese and egg dishes or pizza; Potatoes: Fresh and processed potatoes; Veg: Vegetables; Pasta: Rice, pasta or noodles; Cereal: Breakfast cereals; Fruit: Fresh and processed fruit; Yog: Yoghurt and fromage frais; S-drink: Soft drinks including milk; Conf: Confectionery; Dessert: Ice cream, desserts and cakes; Bisc: Biscuits; Snacks: Crisps, nuts and snacks*

### Dieters


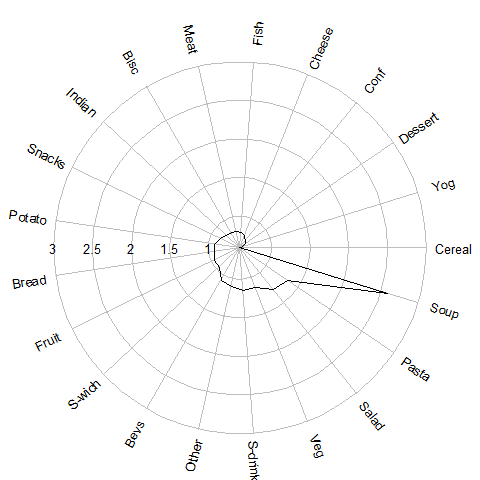


Figure 5. Radial plot showing amount spent per food group for the ‘Dieters’ pattern relative to the mean spending per food group across all clusters (index 1 represents the mean spend for a given food group).

*DEFRA codes have been abbreviated as shown: Indian: Indian, Chinese or Thai food; Meat: Meat & Meat Products; Fish: Fish and fish products; Cheese: Cheese and egg dishes or pizza; Potatoes: Fresh and processed potatoes; Veg: Vegetables; Pasta: Rice, pasta or noodles; Cereal: Breakfast cereals; Fruit: Fresh and processed fruit; Yog: Yoghurt and fromage frais; S-drink: Soft drinks including milk; Conf: Confectionery; Dessert: Ice cream, desserts and cakes; Bisc: Biscuits; Snacks: Crisps, nuts and snacks*

### Dish of the Day


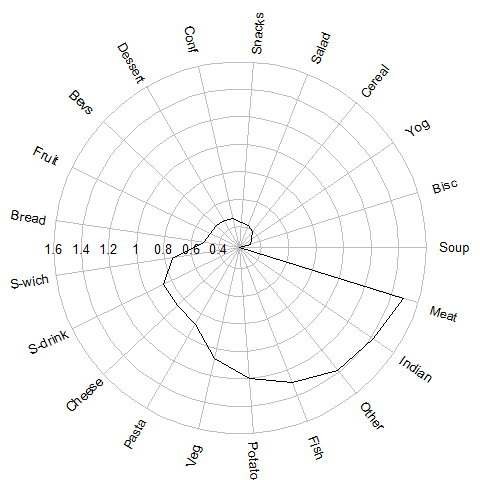


Figure 6. Radial plot showing amount spent per food group for the ‘Dish of the Day’ pattern relative to the mean spending per food group across all clusters (index 1 represents the mean spend for a given food group).

*DEFRA codes have been abbreviated as shown: Indian: Indian, Chinese or Thai food; Meat: Meat & Meat Products; Fish: Fish and fish products; Cheese: Cheese and egg dishes or pizza; Potatoes: Fresh and processed potatoes; Veg: Vegetables; Pasta: Rice, pasta or noodles; Cereal: Breakfast cereals; Fruit: Fresh and processed fruit; Yog: Yoghurt and fromage frais; S-drink: Soft drinks including milk; Conf: Confectionery; Dessert: Ice cream, desserts and cakes; Bisc: Biscuits; Snacks: Crisps, nuts and snacks*

### Grab-and-Go


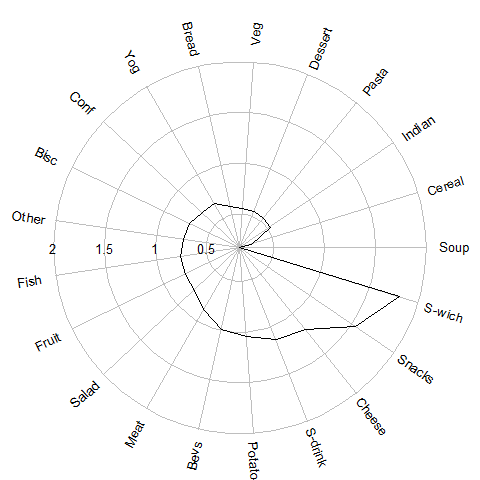


Figure 7. Radial plot showing amount spent per food group for the ‘Grab-and-Go’ pattern relative to the mean spending per food group across all clusters (index 1 represents the mean spend for a given food group).

*DEFRA codes have been abbreviated as shown: Indian: Indian, Chinese or Thai food; Meat: Meat & Meat Products; Fish: Fish and fish products; Cheese: Cheese and egg dishes or pizza; Potatoes: Fresh and processed potatoes; Veg: Vegetables; Pasta: Rice, pasta or noodles; Cereal: Breakfast cereals; Fruit: Fresh and processed fruit; Yog: Yoghurt and fromage frais; S-drink: Soft drinks including milk; Conf: Confectionery; Dessert: Ice cream, desserts and cakes; Bisc: Biscuits; Snacks: Crisps, nuts and snacks*

### Carb Lovers


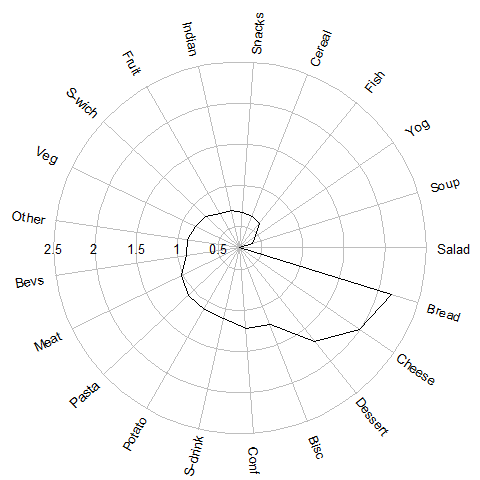


Figure 8. Radial plot showing amount spent per food group for the ‘Carb Lovers’ pattern relative to the mean spending per food group across all clusters (index 1 represents the mean spend for a given food group).

*DEFRA codes have been abbreviated as shown: Indian: Indian, Chinese or Thai food; Meat: Meat & Meat Products; Fish: Fish and fish products; Cheese: Cheese and egg dishes or pizza; Potatoes: Fresh and processed potatoes; Veg: Vegetables; Pasta: Rice, pasta or noodles; Cereal: Breakfast cereals; Fruit: Fresh and processed fruit; Yog: Yoghurt and fromage frais; S-drink: Soft drinks including milk; Conf: Confectionery; Dessert: Ice cream, desserts and cakes; Bisc: Biscuits; Snacks: Crisps, nuts and snacks*

### Snackers


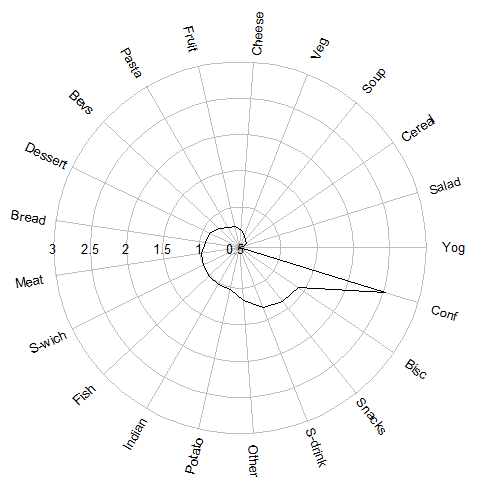


Figure 9. Radial plot showing amount spent per food group for the ‘Snackers’ pattern relative to the mean spending per food group across all clusters (index 1 represents the mean spend for a given food group).

*DEFRA codes have been abbreviated as shown: Indian: Indian, Chinese or Thai food; Meat: Meat & Meat Products; Fish: Fish and fish products; Cheese: Cheese and egg dishes or pizza; Potatoes: Fresh and processed potatoes; Veg: Vegetables; Pasta: Rice, pasta or noodles; Cereal: Breakfast cereals; Fruit: Fresh and processed fruit; Yog: Yoghurt and fromage frais; S-drink: Soft drinks including milk; Conf: Confectionery; Dessert: Ice cream, desserts and cakes; Bisc: Biscuits; Snacks: Crisps, nuts and snacks*

## Supplement 7

Clusters were ranked for healthfulness by:

1. Assigning a variety count indicating the number of food types where spending equalled or exceeded the mean spending for that food type. For example, for the Dish of the Day cluster, 6 food groups had spendings that equalled or exceeded the mean spending for those food groups.
2. Ascribing a variety score = (variety count)/(maximum possible variety count), where maximum possible variety count = 22. For example, for the Dish of the Day cluster the variety score was 6/22 = 0.27.
3. Ascribing Vegetable, Fruit and Salad counts respectively indicating the position of Vegetable, Fruit and Salad food groups in the radial plot. For example, for the Dish of the Day cluster, the Vegetables food group was the 16^th^ food group (from lowest spending to highest) across the 22 DEFRA food groups.
4. Ascribing Vegetable, Fruit and Salad scores = (Vegetable/Fruit/Salad score)/(maximum possible score). For example the Vegetable score for the Dish of the Day cluster was 16/22 = 0.73.
5. Deriving a mean score for variety and Vegetable/Fruit/Salad intake such that the variety score is weighted equally to the Vegetable, Fruit and Salad Scores combined.
6. Ranking the mean scores.

Scores and rankings are shown in the table below.

Table 4. Healthfulness scores and rankings

| Cluster Name | Variety Count | Variety Score | Veg Count | Veg Score | Fruit Count | Fruit Score | Salad Count | Salad Score | Mean Score | Rank |
| --- | --- | --- | --- | --- | --- | --- | --- | --- | --- | --- |
| Dish of the Day | 6 | 0.27 | 16 | 0.73 | 10 | 0.45 | 5 | 0.23 | 0.37 | 4 |
| Omnivores | 13 | 0.59 | 18 | 0.82 | 9 | 0.41 | 12 | 0.55 | 0.59 | 2 |
| Vegetarian | 13 | 0.59 | 12 | 0.55 | 19 | 0.86 | 20 | 0.91 | 0.68 | 1 |
| Carb Lovers | 9 | 0.41 | 10 | 0.45 | 8 | 0.36 | 1 | 0.05 | 0.35 | 6 |
| Dieters | 7 | 0.32 | 8 | 0.36 | 13 | 0.59 | 19 | 0.86 | 0.46 | 3 |
| Snackers | 9 | 0.41 | 5 | 0.23 | 7 | 0.32 | 2 | 0.09 | 0.31 | 7 |
| Grab-and-Go | 5 | 0.23 | 5 | 0.23 | 13 | 0.59 | 14 | 0.64 | 0.36 | 5 |

## Supplement 8

Movement of female and male students between clusters during the three time periods within semester 1 are presented in Table 5 and Table 6 respectively. These tables are visualised using Riverplots in figures 10 and 11. These highlight the difference in behaviours demonstrated by females and males.

Table 5. Cross-tabulation of numbers of female students within dietary clusters during time periods 1-3.

|  |  | Vegetarian | Omnivores | Dieters | Dish of the Day | Grab-and-Go | Carb Lovers | Snackers | % Moving Out |
| --- | --- | --- | --- | --- | --- | --- | --- | --- | --- |
|  |  | **Time Period 2** | | | | | | |  |
| **Time Period 1** | Vegetarian | **60** | 9 | 6 | 0 | 2 | 2 | 2 | 25.9% |
|  | Omnivores | 11 | **42** | 9 | 4 | 1 | 1 | 0 | 38.2% |
|  | Dieters | 8 | 8 | **45** | 3 | 10 | 5 | 13 | 51.1% |
|  | Dish of the Day | 0 | 3 | 2 | **7** | 3 | 4 | 4 | 69.6% |
|  | Grab and Go | 8 | 0 | 9 | 0 | **16** | 2 | 8 | 62.8% |
|  | Carb Lovers | 3 | 1 | 6 | 4 | 2 | **24** | 5 | 46.7% |
|  | Snackers | 2 | 3 | 7 | 9 | 2 | 6 | **40** | 42.0% |
|  | % Moving In | 34.8% | 36.4% | 46.4% | 74.1% | 55.6% | 45.5% | 44.4% |  |
|  |  | **Time Period 3** | | | | | | |  |
| **Time Period 1** | Vegetarian | **50** | 7 | 14 | 1 | 1 | 3 | 5 | 38.3% |
|  | Omnivores | 12 | **34** | 9 | 6 | 1 | 4 | 2 | 50.0% |
|  | Dieters | 12 | 6 | **42** | 7 | 6 | 2 | 17 | 54.4% |
|  | Dish of the Day | 0 | 2 | 6 | **6** | 5 | 2 | 2 | 73.9% |
|  | Grab and Go | 7 | 3 | 7 | 1 | **17** | 3 | 5 | 60.5% |
|  | Carb Lovers | 4 | 3 | 5 | 1 | 6 | **20** | 6 | 55.6% |
|  | Snackers | 3 | 7 | 7 | 7 | 5 | 7 | **33** | 52.2% |
|  | % Moving In | 43.2% | 45.2% | 53.3% | 79.3% | 58.5% | 51.2% | 52.9% |  |
|  |  | **Time Period 3** | | | | | | |  |
| **Time Period 2** | Vegetarian | **60** | 6 | 17 | 0 | 3 | 2 | 4 | 34.8% |
|  | Omnivores | 10 | **39** | 4 | 7 | 0 | 3 | 3 | 40.9% |
|  | Dieters | 13 | 3 | **45** | 4 | 4 | 4 | 11 | 46.4% |
|  | Dish of the Day | 0 | 4 | 5 | **13** | 3 | 0 | 2 | 51.9% |
|  | Grab-and-Go | 4 | 3 | 2 | 0 | **20** | 1 | 6 | 44.4% |
|  | Carb Lovers | 0 | 3 | 7 | 1 | 3 | **27** | 3 | 38.6% |
|  | Snackers | 1 | 4 | 10 | 4 | 8 | 4 | **41** | 43.1% |
|  | % Moving In | 31.8% | 37.1% | 50.0% | 55.2% | 51.2% | 34.1% | 41.4% |  |

Table 6. Cross-tabulation of numbers of male students within dietary clusters during time periods 1-3.

|  |  | Vegetarian | Omnivores | Dieters | Dish of the Day | Grab-and-Go | Carb Lovers | Snackers | % Moving Out |
| --- | --- | --- | --- | --- | --- | --- | --- | --- | --- |
|  |  | **Time Period 2** | | | | | | |  |
| **Time Period 1** | Vegetarian | **6** | 4 | 2 | 0 | 0 | 0 | 1 | 53.8% |
|  | Omnivores | 1 | **26** | 3 | 7 | 2 | 3 | 3 | 42.2% |
|  | Dieters | 2 | 3 | **8** | 1 | 1 | 1 | 4 | 60.0% |
|  | Dish of the Day | 0 | 8 | 3 | **69** | 8 | 4 | 5 | 28.9% |
|  | Grab and Go | 1 | 2 | 6 | 9 | **38** | 13 | 3 | 47.2% |
|  | Carb Lovers | 0 | 7 | 1 | 7 | 6 | **17** | 3 | 58.5% |
|  | Snackers | 1 | 3 | 2 | 5 | 6 | 3 | **26** | 43.5% |
|  | % Moving In | 45.5% | 50.9% | 68.0% | 29.6% | 37.7% | 58.5% | 42.2% |  |
|  |  | **Time Period 3** | | | | | | |  |
| **Time Period 1** | Vegetarian | **5** | 2 | 3 | 0 | 2 | 1 | 0 | 61.5% |
|  | Omnivores | 1 | **28** | 3 | 5 | 3 | 3 | 2 | 37.8% |
|  | Dieters | 1 | 0 | **9** | 3 | 1 | 1 | 5 | 55.0% |
|  | Dish of the Day | 1 | 7 | 4 | **62** | 8 | 8 | 7 | 36.1% |
|  | Grab and Go | 0 | 1 | 6 | 10 | **32** | 14 | 9 | 55.6% |
|  | Carb Lovers | 0 | 8 | 2 | 6 | 2 | **18** | 5 | 56.1% |
|  | Snackers | 1 | 2 | 0 | 7 | 10 | 3 | **23** | 50.0% |
|  | % Moving In | 44.4% | 41.7% | 66.7% | 33.3% | 44.8% | 62.5% | 54.9% |  |
|  |  | **Time Period 3** | | | | | | |  |
| **Time Period 2** | Vegetarian | **5** | 0 | 3 | 1 | 1 | 1 | 0 | 54.5% |
|  | Omnivores | 2 | **35** | 1 | 7 | 3 | 3 | 2 | 34.0% |
|  | Dieters | 1 | 1 | **14** | 3 | 2 | 1 | 3 | 44.0% |
|  | Dish of the Day | 1 | 8 | 2 | **69** | 8 | 7 | 3 | 29.6% |
|  | Grab-and-Go | 0 | 0 | 3 | 6 | **32** | 12 | 8 | 47.5% |
|  | Carb Lovers | 0 | 1 | 2 | 4 | 4 | **23** | 7 | 43.9% |
|  | Snackers | 0 | 3 | 2 | 3 | 8 | 1 | **28** | 37.8% |
|  | % Moving In | 44.4% | 27.1% | 48.1% | 25.8% | 44.8% | 52.1% | 45.1% |  |

##
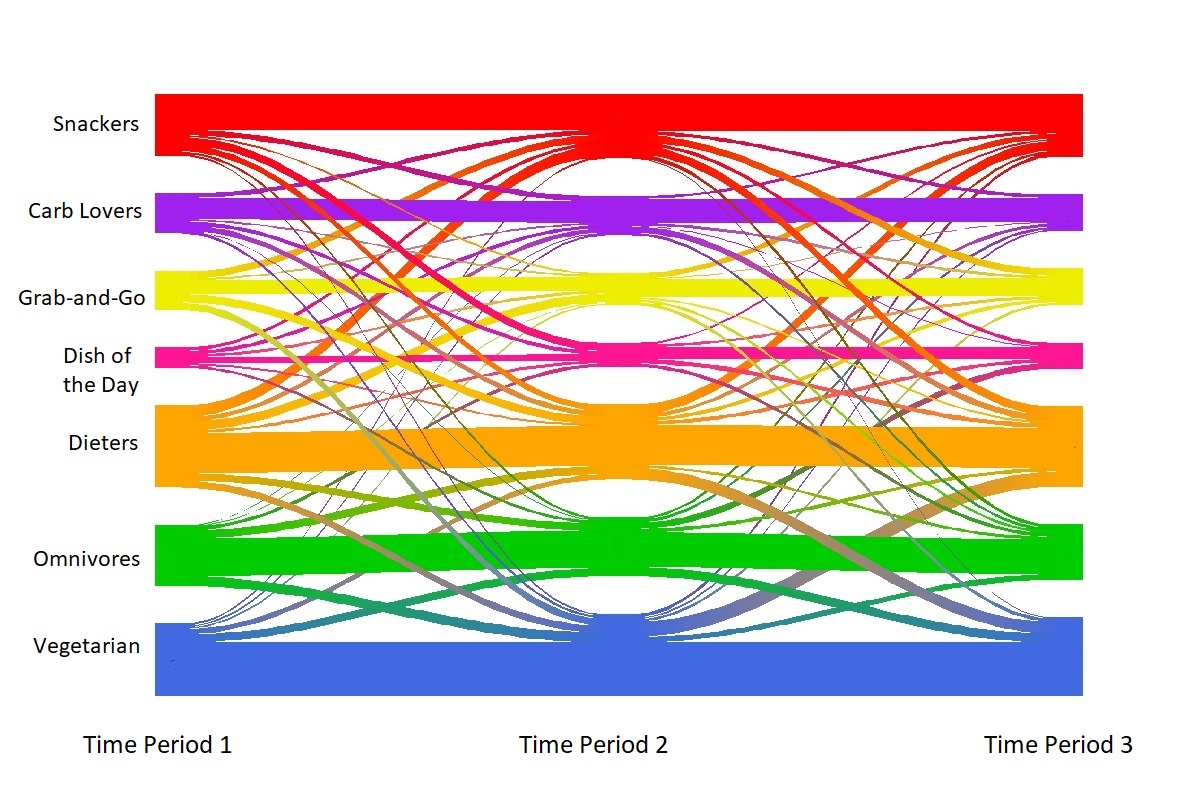


Figure 10. Riverplot showing the flow of female students between dietary clusters at time periods 1-3


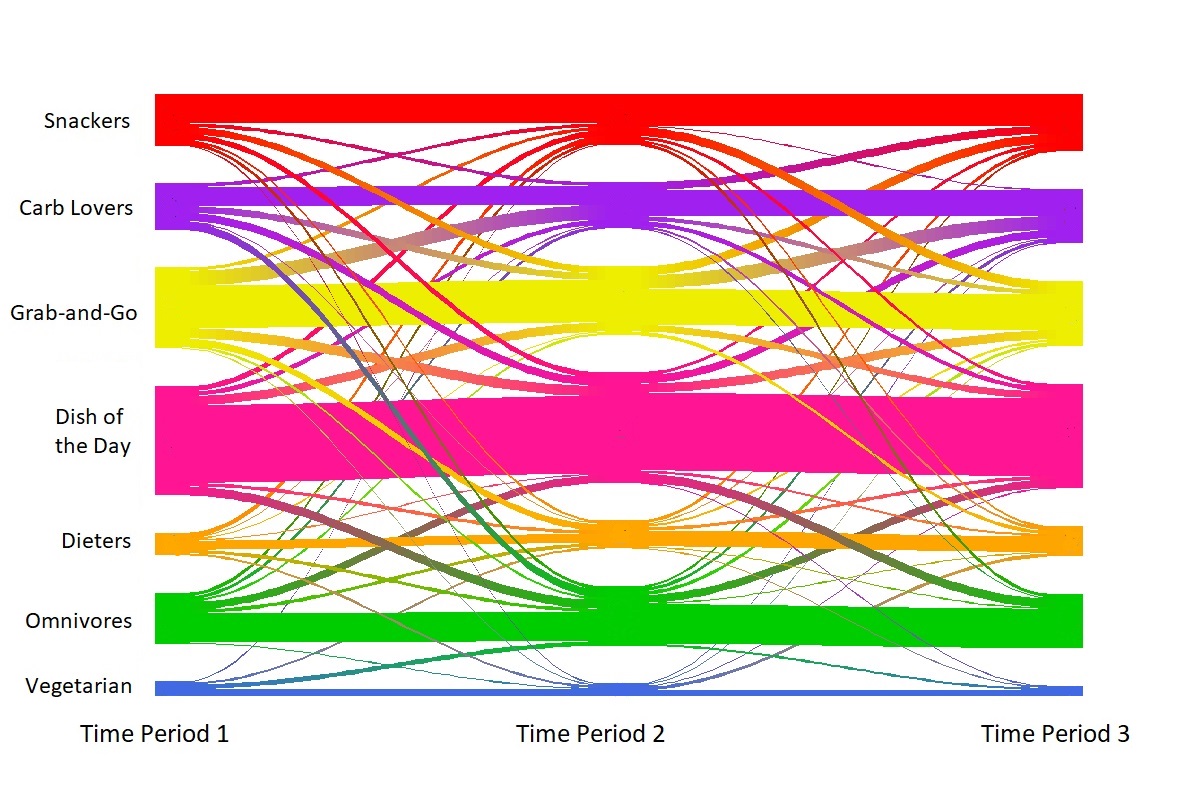


Figure 11 Riverplot showing the flow of male students between dietary clusters at time periods 1-3

## References

DEFRA. 2014. *Household and eating out food & drink codes* [Online]. Available: <https://assets.publishing.service.gov.uk/government/uploads/system/uploads/attachment_data/file/384778/familyfood-method-codes-11dec14.pdf> [Accessed 14 March 2019].

MORRIS, M. A., WILKINS, E., TIMMINS, K. A., BRYANT, M., BIRKIN, M. & GRIFFITHS, C. 2018. Can big data solve a big problem? Reporting the obesity data landscape in line with the Foresight obesity system map. *International journal of obesity*.

UNIVERSITY OF LEEDS. 2019. *The Refectory* [Online]. Available: <https://gfal.leeds.ac.uk/where-to-eat/refectory/#week-2> [Accessed 14 March 2019].
